# Altered Islet Composition and Disproportionate Loss of Large Islets in Patients with Type 2 Diabetes

**DOI:** 10.1371/journal.pone.0027445

**Published:** 2011-11-15

**Authors:** German Kilimnik, Billy Zhao, Junghyo Jo, Vipul Periwal, Piotr Witkowski, Ryosuke Misawa, Manami Hara

**Affiliations:** 1 Department of Medicine, The University of Chicago, Chicago, Illinois, United States of America; 2 Laboratory of Biological Modeling, National Institute of Diabetes and Digestive and Kidney Diseases, National Institutes of Health, Bethesda, Maryland, United States of America; 3 Department of Surgery, The University of Chicago, Chicago, Illinois, United States of America; The University of Hong Kong, Hong Kong

## Abstract

Human islets exhibit distinct islet architecture with intermingled alpha- and beta-cells particularly in large islets. In this study, we quantitatively examined pathological changes of the pancreas in patients with type 2 diabetes (T2D). Specifically, we tested a hypothesis that changes in endocrine cell mass and composition are islet-size dependent. A large-scale analysis of cadaveric pancreatic sections from T2D patients (*n* = 12) and non-diabetic subjects (*n* = 14) was carried out combined with semi-automated analysis to quantify changes in islet architecture. The method provided the representative islet distribution in the whole pancreas section that allowed us to examine details of endocrine cell composition in individual islets. We observed a preferential loss of large islets (>60 µm in diameter) in T2D patients compared to non-diabetic subjects. Analysis of islet cell composition revealed that the beta-cell fraction in large islets was decreased in T2D patients. This change was accompanied by a reciprocal increase in alpha-cell fraction, however total alpha-cell area was decreased along with beta-cells in T2D. Delta-cell fraction and area remained unchanged. The computer-assisted quantification of morphological changes in islet structure minimizes sampling bias. Significant beta-cell loss was observed in large islets in T2D, in which alpha-cell ratio reciprocally increased. However, there was no alpha-cell expansion and the total alpha-cell area was also decreased. Changes in islet architecture were marked in large islets. Our method is widely applicable to various specimens using standard immunohistochemical analysis that may be particularly useful to study large animals including humans where large organ size precludes manual quantitation of organ morphology.

## Introduction

Type 2 diabetes (T2D) is a metabolic disease caused by a relative lack of insulin- mediated control of glucose homeostasis. The gradual progression of T2D hampers determination of the precise onset of the disease. The diagnosis is currently only confirmed when a patient develops chronic hyperglycemia, which is recognized by the patient with various symptoms such as excessive thirst, frequent urination, fatigue, blurred vision, and weight loss. As the onset of T2D is poorly defined, this silent aspect of disease progression has been noted as one of the major obstacles for the treatment of T2D. Levetan et al. have reported that 40% of hospitalized patients with T2D were undiagnosed [1]. It has been estimated that most patients with T2D are not diagnosed until ∼10 years after the disease onset [2].

The unclear pathogenesis leads to incomplete understanding of the disease. While it is well-known that type 1 diabetes results from selective autoimmune destruction of pancreatic beta-cells, there is still considerable debate over the degree of beta-cell loss in T2D in relation to beta-cell dysfunction [3]–[5], which has important clinical implications regarding the treatment of the disease including possible regenerative therapies. The challenge of developing *in vivo* imaging modalities of beta-cell mass in humans is well recognized, and pancreas biopsy is not an option. Therefore, we reasoned that it is critical to obtain maximal information from available autopsy specimens. In fact, it is only recently that cadaveric pancreas specimens have been made more accessible to the scientific community, owing in part to the clinical success of islet transplantation, in which the assessment of the donor pancreas and islet quality is an important issue to improve the clinical outcomes.

Recent immunohistochemistry studies of the human pancreas have demonstrated the distinct islet architecture with more alpha-cells intermingled with beta-cells in humans compared to rodents with the central core of beta-cells with less alpha-cells residing in the periphery [6]–[8]. In interspecies comparative studies, we have shown that such drastic morphological changes occur selectively in large islets (>50–100 µm in diameter) in humans, and similar changes are also observed in mice under conditions of an increased demand for insulin such as pregnancy, obesity and diabetes [9], [10]. In large animals, including humans, a proportionate increase in the pancreas size, islet number and total islet mass compensates for an increased demand for insulin [11], [12]. However, there is no increase in the range of islet sizes in humans compared to that in mice as well as in various other species, where the maximum diameter is around 500 µm [9], [10] suggesting that there are certain regulatory mechanisms that maintain optimal islet sizes in order to ensure their functional properties. The striking plasticity of islet architecture in large islets together with changes in islet size distribution may be used as additional parameters to assess the pathological changes in the pancreas. Collectively, morphological and pathological studies of autopsy pancreatic tissues provide valuable information that may not have been fully explored.

Here we propose to develop a comprehensive evaluation method of autopsy studies in order to better understand the pathophysiology of T2D within the limited sample size and tissues from each specimen. The large-scale analysis quantifies morphology and cellular composition of individual islets. We found a preferential loss of large islets (diameter >60 µm) accompanied by total beta- and alpha-cell area, reduction of beta-cell fraction, increase of alpha-cell fraction, and no change of delta-cell fraction in the large islets. Furthermore, the present study reports the unexpected complexity of pathophysiology of the pancreas, where T2D (from clinical diagnosis and history of patients) does not correlate simply with the degree of deterioration of the pancreas by histology, but may involve a number of factors such as aging, metabolic conditions, and intra-pancreatic fat accumulation. Large-scale data collection combined with automated quantification provides an unbiased baseline to further integrate various environmental signals that affect pathophysiology of the pancreas under metabolic disease states including T2D.

## Materials and Methods

### Human pancreas specimens

The autopsy specimens were collected at the University of Chicago (D1, D2, D6, D7, D9, D12, ND1, ND2, ND4-ND9) and Peninsula Medical School, Plymouth, UK and Royal Infirmary, Glasgow, UK (D3-D5, D8, D10, D11, ND3, ND10-ND14). The use of human tissues in the present study has been approved by the Research Ethics Committee at Peninsula Medical School and Glasgow Royal Infirmary, and the Institutional Review Board at the University of Chicago. The need for consent was waived.

### Immunohistochemistry

Paraffin-embedded sections with 6 µm in thickness were stained with a polyclonal guinea pig anti-porcine insulin primary antibody (DAKO, Carpinteria, CA), a mouse monoclonal anti-human primary glucagon antibody (Sigma-Aldrich, St. Louis, MO), a polyclonal goat anti-somatostatin (Santa Cruz, Santa Cruz, CA) and DAPI (Invitrogen, Carlsbad, CA). The primary antibodies were detected using a combination of DyLight 488, 549, and 649-conjugated secondary antibodies (Jackson ImmunoResearch Lab., West Grove, PA).

### Image capture and quantification

Microscopic images were taken with an Olympus IX8 DSU spinning disk confocal microscope (Melville, NY) with imaging software StereoInvestigator (SI, MicroBrightField, Williston, VT). A modified method of “virtual slice capture” [13]–[15] was used. Briefly, the SI controls a XYZ-motorized stage and acquires consecutive images and creates a high-resolution montage composed of images obtained from multiple microscopic fields of view. The entire tissue section was captured as “a virtual slice” using a 10x objective. Each virtual slice taken at four fluorescent channels were further merged into one composite. Quantification of cellular composition (i.e. each area of beta-, alpha-, and delta-cell populations, and islet area by automated contouring of each islet) was carried out using a macro written for ImageJ (http://rsbweb.nih.gov/ij/). To measure coordinates of each islet-cell type, the DAPI fluorescent signals were converted to 8-bit masks and watershed to obtain masks of individual nuclei. The pixels surrounding nuclei masks were quantified with respect to each endocrine hormone staining (i.e. insulin, glucagon, or somatostatin) to identify which hormone was most prevalent around each nucleus and to record islet-cell coordinates. DAPI signals outside of islets were not included. Mathematica (Wolfram Research, Champaign, IL) was used for mathematical analyses.

### Characterization of islet architecture

The image analysis identifies individual islets on pancreatic sections, and obtains center coordinates of every endocrine cell in each islet. This allows us to calculate probabilities of contact between cell types. For example, the probability, *Pαα*, quantifies how many alpha cells contact with alpha cells in an islet. To identify a contact of two cells (i.e. neighborhood cells), we calculated a distance between center coordinates of the two cells. If the distance is less than a given threshold distance, they are defined as a neighborhood. Here the threshold distance is set as 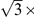
cell diameter that is the distance to the second nearest-neighboring site in hexagonal lattice. We have also defined neighborhood with a different threshold, 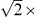
cell diameter, which is the distance to the second nearest-neighboring site in square lattice. We checked that the small differences of the two threshold distances did not change the probabilities of contact between cell types. Here it is not trivial to determine ‘cell diameter’ from the only given information of center coordinates of cells within an islet. We calculated distances between every pair of nearest-neighboring cells on each islet. We assume that the mean nearest-neighboring distance (20 ± 12 µm) corresponds to cell diameter because the center-to-center distance between (perfectly) contacting cells is equal to cell diameter. If the neighborhood of every cell in an islet is identified as described, it is straightforward to calculate numbers of alpha-alpha, alpha-beta, alpha-delta, beta-beta, beta-delta, and delta-delta contacts within the islet. Then, the probabilities of contact between specific cell types are just the contact number between them normalized by total number of contact between cells. These quantities represent islet architecture. For example, suppose we have a certain number of alpha and beta cells. If contacts between homotypic cells were preferable, we would observe more frequent alpha-alpha and beta-beta contacts compared with random cell arrangement. Here the probabilities of contact between cell types for the random cell arrangement (*Pαα*, *Pββ*, *Pαβ*) are theoretically estimated as (*Pα* ×*Pα*, *Pβ* ×*Pβ*, *Pα* ×*Pβ* +*Pβ* ×*Pα*), for a given cellular compositions (*Pα*, *Pβ*).

### Statistical analysis

Data are expressed as mean ± SEM. Statistical analyses were performed using Student's *t* test. Differences were considered to be significant at *P*< 0.05.

## Results

### Large-scale capture and computer-assisted semi-automated analysis of the whole tissue section

**Figure 1:**
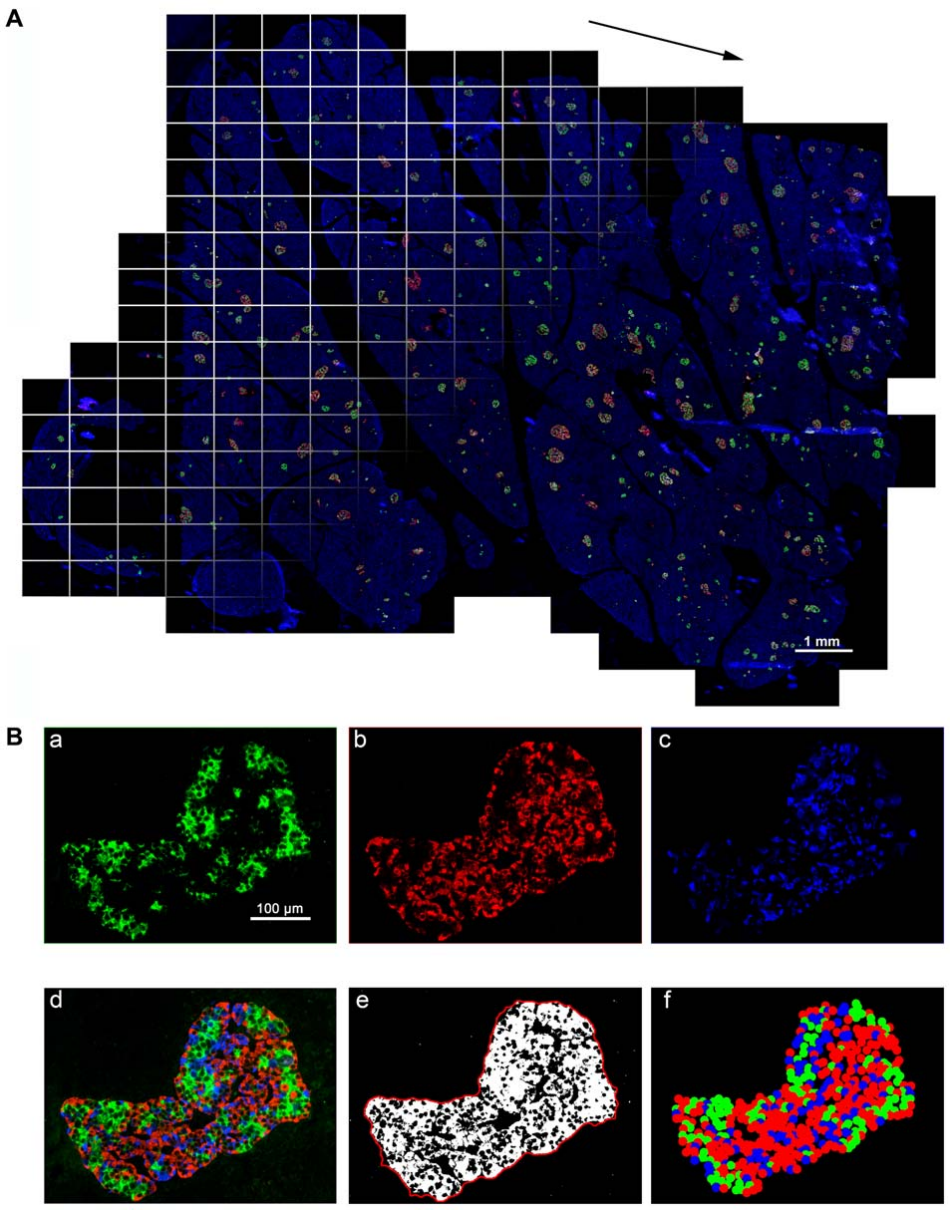
Large-scale capture and computer-assisted semi-automated analysis of the whole tissue section. **A**: Virtual slice view of a human pancreatic section (ND11) immunostained for insulin (green), glucagon (red), somatostatin (white) and nuclei (blue). A series of contiguous images of a specimen is collected (illustrated as boxed panels) and merged into a single image montage (i.e. virtual slice; arrowed). A composite is made by merging four overlapping virtual slice images. **B**: Views of each channel showing cellular composition. **a.** beta-cells, **b.** alpha-cells, **c**. delta-cells, and **d.** composite of all three endocrine cells. Note that there is no overlap among the endocrine cell fractions. Corresponding converted 8-bit masks after automatic thresholidng are shown in **e**. A contour shown in e (a red line) is used to measure islet area, which includes non-labeled area (e.g. capillaries). Based on the captured center coordinates of each cell type within the given islet, its architecture is reconstructed in **f**.

Islet size distribution and cellular composition within whole human pancreatic tissue sections from subjects with T2D were examined using large-scale analyses that we have developed. Specimens from those without a history of diabetes were also included for comparison. The detailed information on the donors (T2D, *n* = 12 and non-diabetic, *n* = 14) is summarized in Table 1. The cases are listed by ascending age in each group. The method to capture the entire distribution of islets within the whole section and computer-assisted semi-automated data analyses are schematically described in Fig. 1. We have applied a method to quantify the distribution of islets in the intact mouse pancreas, which has been described in [13], [14], and also shown in a visual article [15]. The modification of our method used in this study is to quantify the islet size distribution along with information on beta-, alpha-, and delta-cell composition within each islet as well as nuclei (DAPI) by capturing fluorescence on four channels over the entire section. Figure 1A shows a combined projection of all the channels of immunohistochemical staining of a human pancreas section. Note that the image including DAPI nuclei staining shows the entire pancreas section. The computer-based automated method identifies every islet with a specific identification number and provides the precise quantification of cellular composition (i.e. the area of beta-, alpha-, and delta-cells within a given islet; Fig. 1B).

**Table 1:** Sample information.

ID	Age	Sex	COD*/Clinical Information
D1	38	F	Stroke.
D2	42	M	Stroke.
D3	65	F	Diabetic nephropathy.
D4	66	F	Renal abscess.
D5	67	F	Gastric cancer.
D6	67	F	Intracranial bleeding. 7.5% HbA1c.
D7	71	F	Head trauma. T2D for 15 years, on Insulin for 3 years, high cholesterol, high BP. No alcohol, no cigarettes.
D8	71	M	Diabetic for 5yr; leg gangrene.
D9	72	F	Treated T2D for 15 years; HbA1c: 6.7%.
D10	73	M	Meningitis.
D11	75	F	Enteritis.
D12	81	M	Anoxia. HbA1c: 5.5%.
ND1	15	M	Anoxia.
ND2	21	M	Head trauma.
ND3	24	M	Trauma.
ND4	41	F	Stroke
ND5	45	F	Stroke.
ND6	47	M	Stroke. Cigarette use, alcohol use, IV drug user; heroin daily for 10 years, cocaine weekly for 5 years.
ND7	51	F	Anoxia.
ND8	51	M	Kidney stones. Stroke.
ND9	53	M	Stroke.
ND10	63	F	Pulmonary thromboembolism.
ND11	63	F	Esophageal cancer.
ND12	68	M	Ruptured esophagus.
ND13	73	M	Stomach cancer.
ND14	81	M	Colon cancer.

*COD: cause of death.

### Morphology of the pancreas and islets

**Figure 2:**
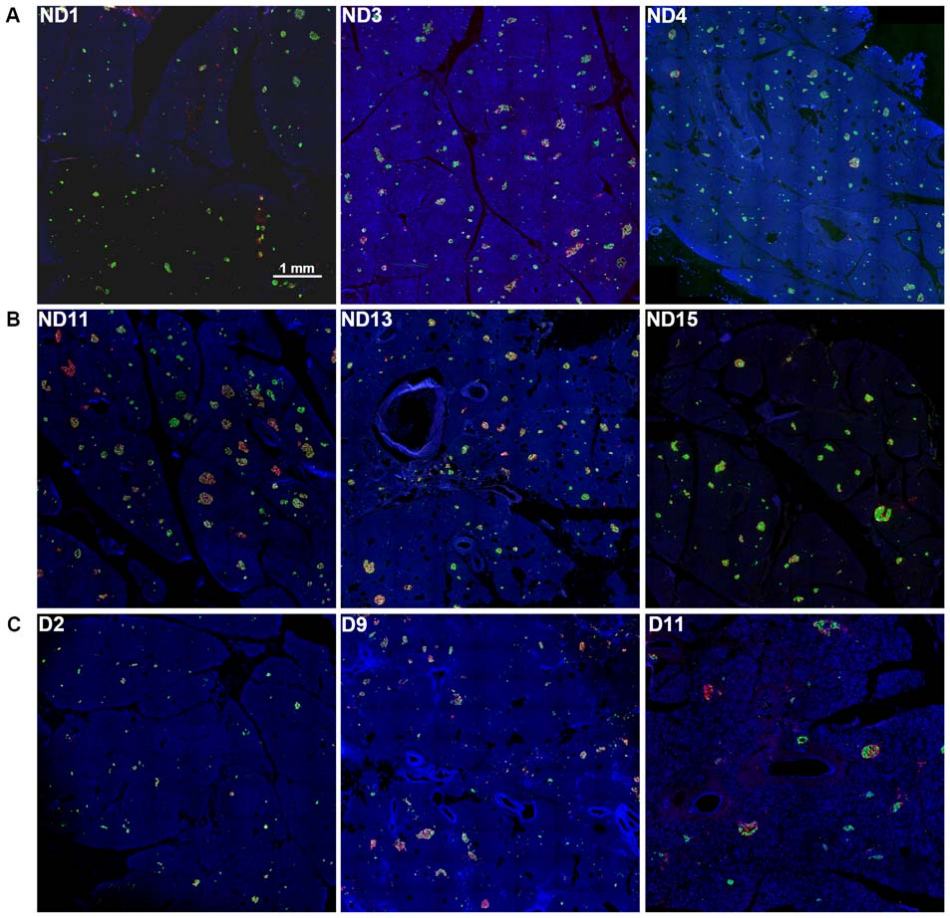
Representative pancreas images. **A:** Pancreatic sections immunostained for insulin (green), glucagon (red), somatostatin (white) and nuclei (blue) of relatively young and healthy subjects. ND1: 15-yr, ND3: 24-yr, and ND4: 41-yr. **B:** Non-diabetic and aged subjects. ND11: 63-yr, ND13: 73-yr, and ND15: 81-yr. **C:** Subjects with T2D. D2: 42-yr, D9: 72-yr, and D11: 75-yr.

Representative pancreas images shown in Fig. 2 are from roughly selected specimens grouped by available clinical information: (1) relatively young and healthy subjects (Fig. 2A: ND1, ND3, ND4); (2) non-diabetic elderly subjects (Fig. 2B: ND11, ND13, ND15); and (3) subjects with T2D (Fig. 2C: D2, D9, D11). Note that partial regions of each whole section are shown in the same scale for comparison with reasonable resolutions, due to the large size (from 2 to 5 GB) of the original images. While, at a glance, there appears to be an overall difference in total islet mass among the three groups, the detailed analysis described below demonstrates the complexity of beta-cell loss and clinical manifestations.

**Figure 3:**
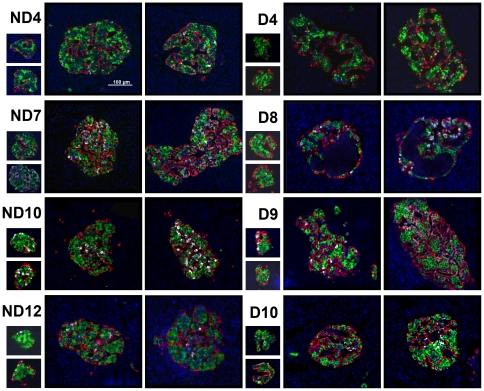
Morphology of randomly selected islets. ND4, ND7, ND10, ND12: specimen from non-diabetic subjects. D4, D8-D10: specimens from subjects with T2D. All listed by the ascending order of age in each group.

Several large and small islets randomly selected from each section are presented in Fig. 3 (non-diabetic: ND4, ND7, ND10, ND12 and T2D: D4, D8-D10; listed in ascending order of age in each group). Islet architecture is well retained in small islets in all specimens, whereas large islets exhibit various changes from compacted endocrine cell arrangement (ND4, ND7, ND10, ND12) to relatively sparse architecture (D4, D10), then to that of a population of cells lacking cytosolic hormones (D9). As an extreme example, a few large islets in the D8 pancreas contained a cyst with necrotic materials that was not stained with DAPI.

### Loss of beta- and alpha-cells and altered morphology in large islets in T2D

**Figure 4:**
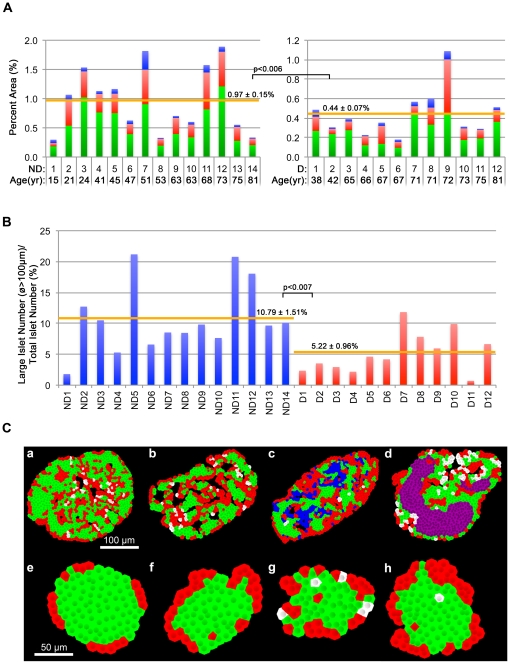
Endocrine cell mass and characteristics of islet architecture. **A:** Inter-subject comparison. Total islet cell composition (beta-cells in green, alpha-cells in red, and delta-cells in blue) in individual non-diabetic subjects (a) and T2D patients (b). **B:** Morphological changes observed in large islets. Representative islets are pseudo-colored models of actual islets based on immunohistochemical images composed of beta-cells (green), alpha-cells (red), and delta-cells (white). From left to right: compacted endocrine cell arrangement, relatively sparse architecture, the presence of a population of cells lacking cytosolic hormones (blue), and cyst formation containing necrotic materials (purple). Corresponding small islets from the same sections in **a** to **d** are shown in **e** to **h**.

Total islet cell composition (beta-cells in green, alpha-cells in red and delta-cells in blue) in each specimen is compared between non-diabetic (Fig. 4A.a) and T2D (Fig. 4A.b) subjects. Total islet area was significantly decreased in T2D compared to non-diabetic subjects (0.5 ± 0.08% and 1.08 ± 0.18%, respectively, *P*<0.008). This decrease was due to total beta-cell loss (0.29 ± 0.04% and 0.65 ± 0.11%, respectively. *P*<0.006) as well as total alpha-cell loss (0.16 ± 0.04% and 0.35 ± 0.06%, respectively, *P*<0.02). There was a decrease in total delta-cell area, but the difference was not statistically significant (0.04 ± 0.01% and 0.09 ± 0.03%, respectively, *P* = 0.13). Comparison of the ratio of large islets (>100 µm in diameter, i.e. the clinically relevant size of islets) to the total number of islets showed significant differences between non-diabetic and T2D subjects (11.08 ± 0.02% and 5.22 ± 0.01%, respectively, *P*<0.007), confirming a preferential loss of large islets by a simple comparison of the mean values of individual subjects of the two groups (Fig. 4B). Accompanied with significant changes in cellular composition in large islets, the presence of a few large islets with altered islet morphology (Fig. 3) may provide landmarks for the progression of overt diabetes (Fig. 4C). While large human islets generally show the compacted endocrine cell arrangement displaying alpha- and delta-cells being intermingled with beta-cells (Fig. 4C.a: beta-cells shown in green, alpha-cell in red and delta-cells in white), specimens from subjects with T2D showed altered islet morphology: relatively sparse architecture (Fig. 4C.b); the presence of a population of cells lacking cytosolic hormones (blue; Fig. 4C.c); and cyst formation containing necrotic materials (purple; Fig. 4C.d). No marked morphological changes were observed in small islets (Fig. 4C. e-f).

### Islet size distribution and cellular compositions

**Figure 5:**
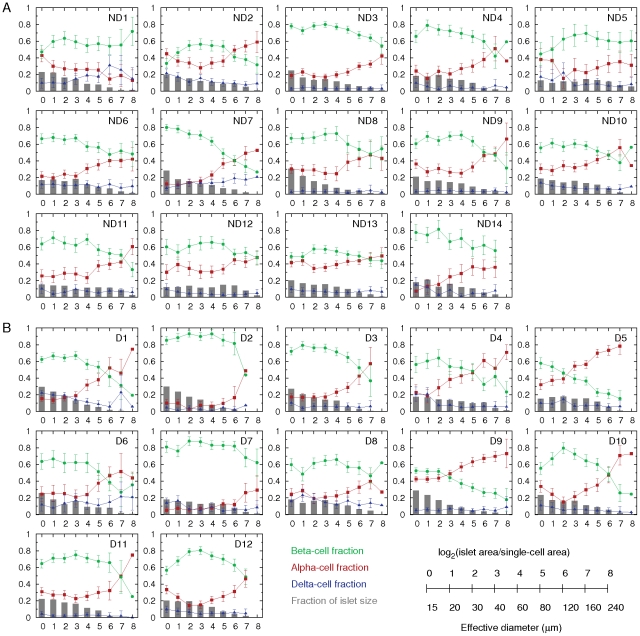
Individual islet size distribution and cellular compositions. **A:** In non-diabetic subjects (ND1–ND14), relative frequency of islet size (gray bar) and ratios of alpha (red), beta (green), and delta (blue) cells within islets were plotted against islet size; means ± SEM. **B:** Results in T2D patients (D1–D12). Note that islet size is presented as a logarithmic scale considering the high number of small islets and the low number of large islets. In addition, we divided islet areas by the single-cell area, 170 µm^2^ [29], to make them as dimensionless values representing the number of cells in a given islet area. The conversion between logarithmic islet area (logarithmic) and effective diameter (µm) is shown.

**Figure 6:**
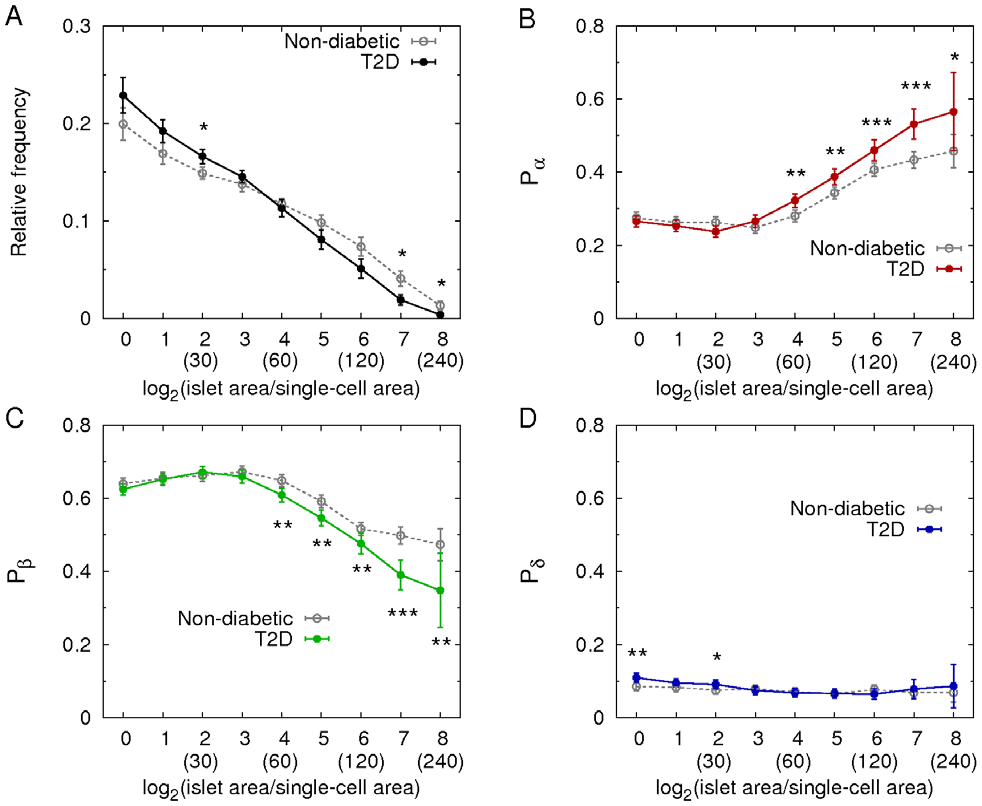
Changes of islet size and cellular compositions in T2D. **A:** Mean relative frequencies of islet sizes and cellular compositions (**B:** alpha-cell fraction, **C:** beta-cell fraction, and **D:** delta-cell fraction) depending on islet size in non-diabetic (gray; 8903 islets with n = 14) and T2D (color; 7929 islets with n = 12) subjects (mean ± SEM). The numbers in parentheses represent effective islet diameters (µm) corresponding to the given logarithmic dimensionless islet areas. Student's *t*-test compared the results between non-diabetic and T2D subjects at each size bin with *P<0.05, **P<0.001, and ***P<0.0001.

We further analyzed morphology of individual islets, and examined their size distributions and cellular compositions, depending on islet size, in non-diabetic (Fig. 5A) and T2D (Fig. 5B) subjects. The results showed a large variation between individuals. However, we were able to see significant differences of islet morphology between the two groups. First, the relative frequencies of large islets (>60 µm in diameter) were decreased in T2D patients (Fig. 6A), confirming the preferential loss of large islets in T2D (see Fig. 4B). In particular, larger islets (>160 µm) showed more significant decrease. Note that while islets ranging from 50 to 250 µm constitute only 10% of total number of islets, they consist of 80% of the total islet area. Therefore, the significant reduction in the area ratio of islets to pancreas in T2D resulted from the preferential loss of the large islets. The most severe cases, D4 and D6, showed overtly reduced islet area ratios, 0.22% and 0.19%, respectively, compared with the islet area ratio of non-diabetic subjects examined (1.08 ± 0.18%). It is noted that D9 had a larger islet area ratio of 1.2%. The donor was a 72-yr old female with 15-yr history of diabetes, which was relatively well controlled (HbA1c = 6.7%). Second, the composition of beta- (green), alpha- (red), and delta- (blue) cells in islets was approximately 6∶3∶1 (Fig. 6B-D). In detail, however, the cellular composition depended on islet size. The proportion of alpha-cells increased with islet size, while the proportion of beta-cells reciprocally decreased with islet size. This finding is consistent with previous reports [16]–[18]. Thus a larger islet had more alpha-cells and less beta-cells. On the other hand, the proportion of delta-cells was independent from islet size. In addition to the general features of cellular compositions in islets, we found significant changes in the cellular compositions in T2D. In large islets (>60 µm), beta-cell fraction decreased, while alpha-cell fraction reciprocally increased, suggesting a preferential loss of beta-cells. Delta-cell fraction did not change in the large islets.

### Islet architectures

**Figure 7:**
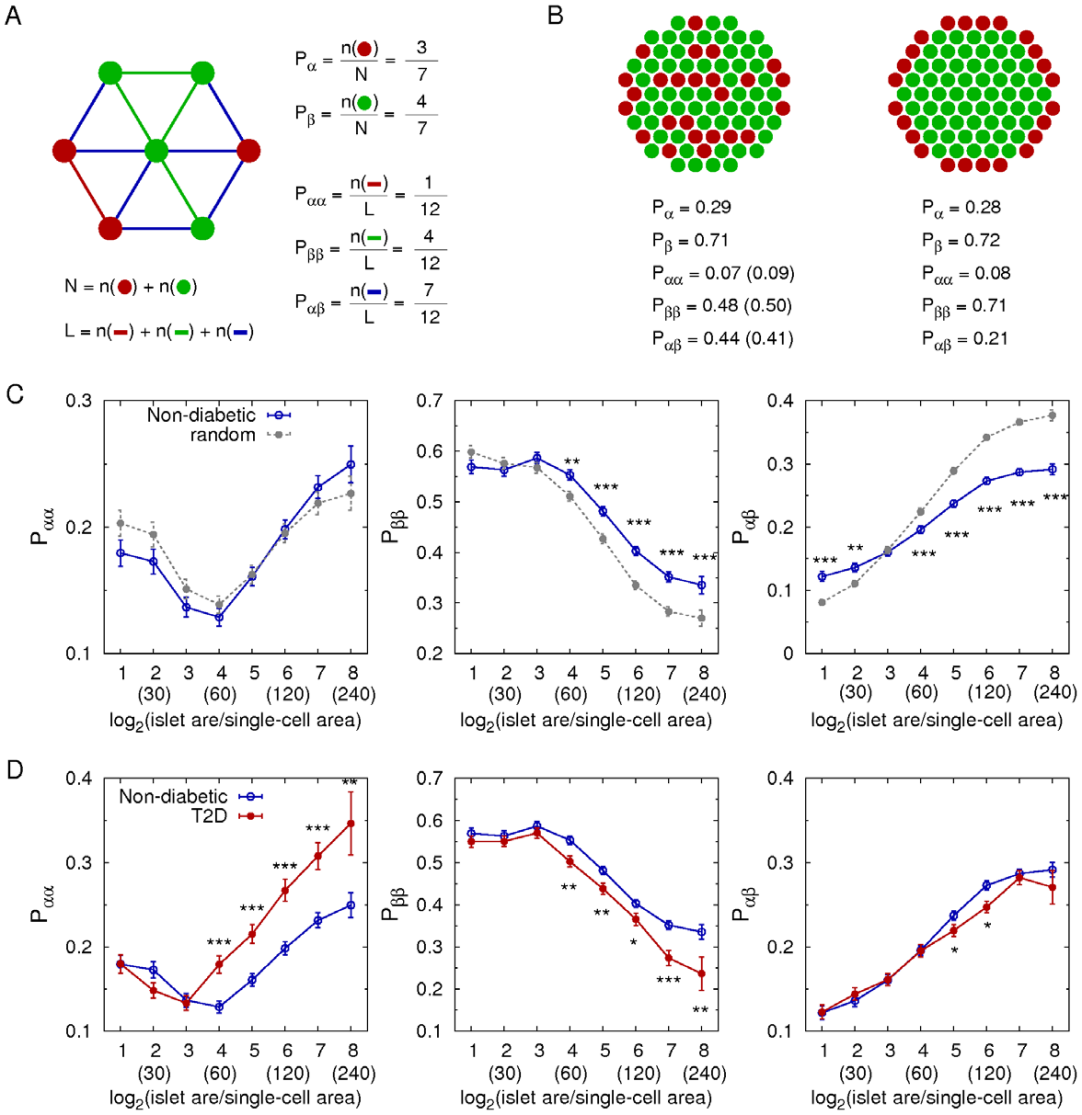
Changes of islet architecture in T2D. **A:** A simple example to calculate cellular compositions and probabilities of contact between cell types in an islet consisting of 3 alpha- (red) and 4 beta- (green) cells. Lines represent contacts between neighboring cells. **B:** two examples of distinctive architectures: random mixture of cells (left) and a regular structure (right) where cellular composition is the same (alpha:beta = 3∶7). The numbers in parentheses are the probabilities of contact between cell types in the random cell mixture, which are theoretically estimated as (*Pαα*  = *Pα* ×*Pα*, *Pββ*  = *Pβ* ×*Pβ*, *Pαβ*  = *Pα* ×*Pβ* +*Pβ* ×*Pα*). **C:** Probabilities of alpha-alpha, beta-beta, and alpha-beta cell contacts in non-diabetic subjects (blue; 6886 islets with n = 14). Those were compared with the theoretical estimation for random mixtures of cells (gray) where cellular compositions were the same with the individual islets in non-diabetic subjects (mean ± SEM). D: Comparison between non-diabetic (blue) and T2D (red; 5359 islets with n = 12) subjects. Note that scattered single endocrine cells are excluded in the calculation because contacting cells does not exist for them. The numbers in parentheses represent effective islet diameters (µm) corresponding to the given logarithmic dimensionless islet areas. Student's *t*-test compared the results between non-diabetic and T2D subjects at each size bin with *P<0.05, **P<0.001, and ***P<0.0001.

**Figure 8:**
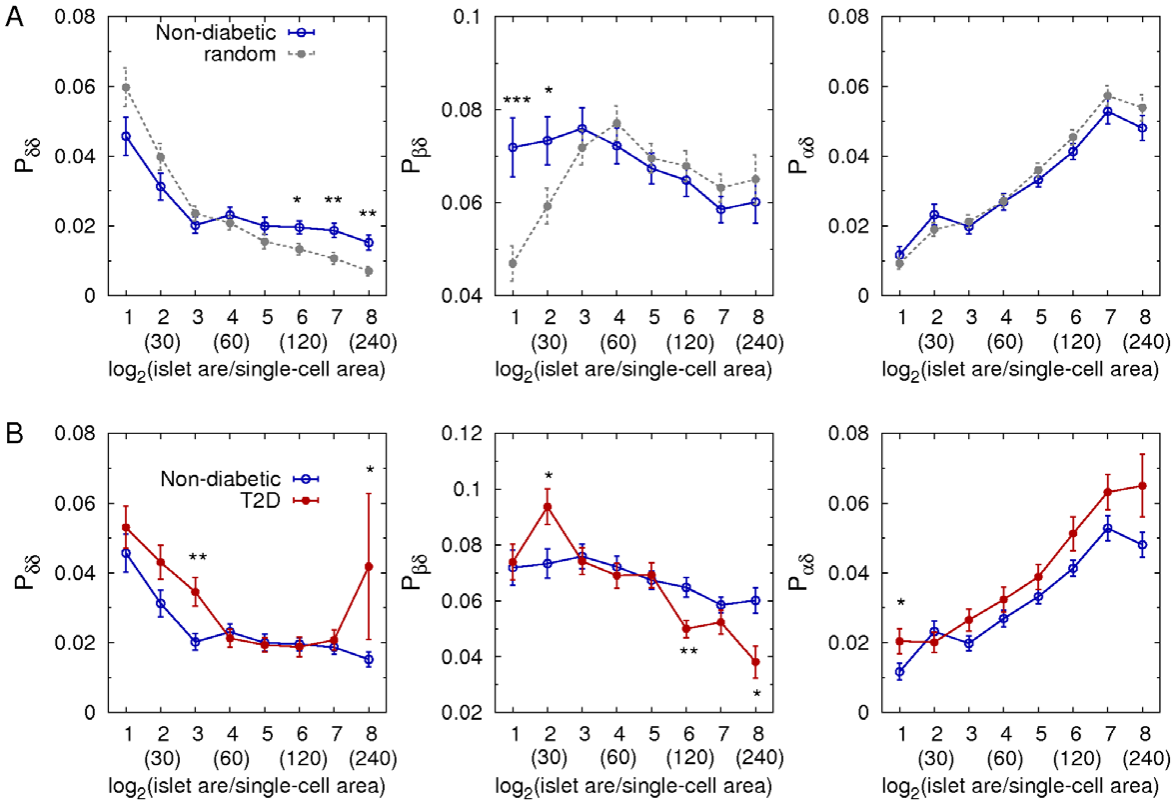
Cellular arrangement of delta-cells. **A:** Probabilities of delta-delta, delta-beta, and delta-alpha cell contacts in non-diabetic subjects (blue; 6886 islets with n = 14). Those were compared with the theoretical estimation for random mixtures of cells (gray) where cellular compositions were the same with the individual islets in non-diabetic subjects (mean ± SEM). B: Comparison between non-diabetic (blue) and T2D (red; 5359 islets with n = 12) subjects. Note that scattered single endocrine cells are excluded in the calculation because contacting cells does not exist for them. The numbers in parentheses represent effective islet diameters (µm) corresponding to the given logarithmic dimensionless islet areas. Student's *t*-test compared the results between non-diabetic and T2D subjects at each size bin with *P<0.05, **P<0.001, and ***P<0.0001.

To quantify cellular arrangement in islets, we calculated the probabilities of contact between cell types as schematically described in Fig. 7A. These can be a relevant measure of islet architectures in the context of paracrine interactions between neighboring cells. For example, we compared a random islet structure with a regular islet structure with the same cellular composition (Fig. 7B). The probabilities of contact between cell types successfully distinguished two completely different structures. First, we compared the real architecture of individual islets in non-diabetic subjects with a random cell mixture while keeping the same cellular composition. Note that we can theoretically estimate the probabilities of contact between cell types in the random cell mixture, given numbers of each cell type (see Materials and Methods). The human islets showed more frequent beta-beta cell contacts, but less frequent alpha-beta cell contacts, compared with the random cell mixture (Fig. 7C). This demonstrated that human islets were not a random mixture of endocrine cells but had a characteristic structure. Second, we compared the islet architectures between non-diabetic and T2D subjects (Fig. 7D). In general, larger islets have more alpha-alpha and alpha-beta cell contacts and less beta-beta cell contacts than smaller islets, reflecting that alpha-cells are more abundant in larger islets. In T2D, the preferential loss of beta-cells in the large islets (>60 µm in diameter) resulted in relative increase of alpha-cell fraction and decrease of beta-cell fraction. This altered cellular composition lead to increased contacts between alpha-cells and decreased contacts between beta-cells. Furthermore, the probability of contact between alpha- and beta-cells decreased in T2D, because decreased beta-cell fraction was slightly dominant in the reciprocal change of alpha- and beta-cell fraction. In addition, delta-delta, delta-beta, and delta-alpha cell contacts also had different patterns from the random cell mixture (Fig. 8A). T2D subjects showed increased delta-delta, decreased delta-beta, and increased delta-alpha cell contacts, compared with non-diabetic subjects (Fig. 8B), which was consistent with decreased beta-cell fraction and increased alpha-cell fraction in large islets. These quantifications explicitly showed that the altered cellular compositions in T2D lead to the changes in cellular arrangement in large islets.

## Discussion

The etiology of T2D implicates peripheral resistance to insulin-mediated glucose uptake that results in an increased demand for insulin to control circulating glucose. The stress of meeting this demand for insulin is followed by gradual loss of proper beta-cell function and sufficient beta-cell mass [4], [5], [19], [20], [21]. When the demand for insulin exceeds the compensatory mechanisms of the body, it leads to chronic hyperglycemia, which is defined as diabetes mellitus. Currently, there are no means available for quantifying beta-cell mass *in vivo* in humans. Studies of the endocrine pancreas in T2D have largely relied on autopsies. Furthermore, clinical information is often limited and variable. Particularly in the case of patients with T2D, determining the precise onset of the disease is unrealistic due to the nature of its gradual progression. Finally, precise comparative studies are hampered by the difficulty of determining a control group in human populations. In the present study, we aimed to evaluate the pathophysiology of T2D within the limitations of the paucity of autopsy specimens and clinical information.

We have developed a large-scale image analysis that can semi-automatically quantify islet morphology and architecture in the whole pancreatic section. The method provides representative islet size distribution. Furthermore, high quality image capturing with computer-aided focusing over hundreds of optical panels that cover a whole section (which are subsequently merged into a single montage) allows us to quantitatively analyze cellular composition of each and every islet. In subjects with T2D, total beta-cell area was significantly reduced as consistent with previous reports [21]–[26]. In the present study, we found a preferential loss of large islets (diameter >60 µm) and altered endocrine cell composition with a reciprocal change of a decrease in beta-cell fraction and an increase of alpha-cell fraction. However, total alpha-cell area was decreased along with that of beta-cells. This is contrary to several previous studies that reported alpha-cell expansion in T2D patients [21], [22], [24], which may be due to sampling bias by selecting specific regions with large islets. A recent study by Henquin and Rahier on the other hand found that alpha-cell mass was identical between T2D patients and non-diabetic subjects [18]. This difference may be due to the method used in their study (100–300 islets/section by point counting at X400 magnification), whereas we quantify an average of >700 islets/section by computer-assisted automated measurement. The important implication from these studies is that the relative hyperglucagonemia often reported in T2D patients is not due to alpha-cell expansion. Our automatic quantification of about 20,000 individual islets clearly showed that human islets were not a random mixture of endocrine cells, but that beta-cells preferred to contact with each other as in rodent islets. This is relevant to islet physiology that the coupling between beta-cells is necessary to generate robust electrical activity for efficient insulin secretion [27], [28]. Thus, the reduced beta-beta cell contacts observed in T2D may reduce essential couplings between beta-cells resulting in inefficient insulin secretion. Our analysis has also shown that higher alpha-cell ratios are only observed in large islets and alpha-cell ratio increases with islet size, which is consistent with previous studies by us [8]–[10] and others [17], [18]. In addition to the higher alpha-cell ratio, the probability of contact between alpha- and beta-cells indeed increased with islet size, suggesting that the interaction between alpha- and beta-cells may play an important role in larger islets. We propose that the analysis on beta-cell mass should be conducted in the context of isle size distribution. When adjacent tissue sections are available, the automated analysis can also be applied to examine islet amyloid polypeptide (IAPP) deposits, inflammation, necrosis, and replication with specific markers.

The large-scale analysis of human pancreas tissues described in the present study can immediately be adopted for the post-evaluation of the clinical outcomes of islet transplantation by reserving a piece of pancreas tissue at the time of procurement, an on-going project that we have initiated at the Pancreatic Islet Isolation Laboratory at the University of Chicago. A possible cohort study at multiple medical centers including re-evaluation of existing samples in the field may help establish a universal scoring method to evaluate islet quality for transplantation, and further define the progression of diabetes that should lead to a better understanding of the pathophysiology of the disease.

## References

[1] Levetan CS, Passaro M, Jablonski K, Kass M, Ratner RE (1998). Unrecognized diabetes among hospitalized patients.. Diabetes Care.

[2] Harris MI, Eastman RC (2000). Early detection of undiagnosed diabetes mellitus: a US perspective.. Diabetes Metab Res Rev.

[3] Matveyenko AV, Butler PC (2008). Relationship between beta-cell mass and diabetes onset.. Diabetes Obes Metab.

[4] Marchetti P, Lupi R, Del Guerra S, Bugliani M, Marselli L (2010). The beta-cell in human type 2 diabetes.. Adv Exp Med Biol.

[5] Marchetti P, Dotta F, Lauro D, Purrello F (2008). An overview of pancreatic beta-cell defects in human type 2 diabetes: implications for treatment.. Regul Pept.

[6] Brissova M, Fowler MJ, Nicholson WE, Chu A, Hirshberg B (2005). Assessment of human pancreatic islet architecture and composition by laser scanning confocal microscopy.. J Histochem Cytochem.

[7] Cabrera O, Berman DM, Kenyon NS, Ricordi C, Berggren PO (2006). The unique cytoarchitecture of human pancreatic islets has implications for islet cell function.. Proc Natl Acad Sci U S A.

[8] Kharouta M, Miller K, Wojcik P, Kilimnik G, Dey A (2009). No mantle formation in rodent islet – the prototype of islet revisited.. Diabetes Res Clin Pract.

[9] Kim A, Miller K, Jo J, Wojcik P, Kilimnik G (2009). Islet architecture – a comparative study.. Islets.

[10] Steiner DJ, Kim A, Miller K, Hara M (2010). Pancreatic islet plasticity: interspecies comparison of islet architecture and composition.. Islets.

[11] Bonner-Weir S (1994). Regulation of pancreatic beta-cell mass in vivo.. Recent Prog Horm Res.

[12] Montanya E, Nacher V, Biarnés M, Soler J (2000). Linear correlation between beta-cell mass and body weight throughout the lifespan in Lewis rats: role of beta-cell hyperplasia and hypertrophy.. Diabetes.

[13] Kilimnik G, Kim A, Jo J, Miller K, Hara M (2009). Quantification of pancreatic islet distribution in situ in mice.. Am J Physiol Endocrinol Metab.

[14] Miller K, Kim A, Klimnik G, Jo J, Moka U (2009). Islet formation during neonatal development.. PLoS One.

[15] Kim A, Kilimnik G, Guo C, Sung J, Jo J (2011). Computer-assisted large-scale visualization and quantification of pancreatic islet mass, size distribution and architecture.. J Vis Exp.

[16] Yoon KH, Ko SH, Cho JH, Lee JM, Ahn YB (2003). Selective beta-cell loss and alpha-cell expansion in patients with type 2 diabetes mellitus in Korea.. J Clin Endocrinol Metab.

[17] Bosco D, Armanet M, Morel P, Niclauss N, Sgrol A (2010). Unique arrangement of alpha- and beta-cells in human islets of Langerhans.. Diabetes.

[18] Henquin JC, Rahier J (2011). Pancreatic alpha cell mass in European subjects with type 2 diabetes.. Diabetologia.

[19] Prentki M, Nolan CJ (2006). Islet beta cell failure in type 2 diabetes.. J Clin Invest.

[20] Matveyenko AV, Butler PC (2008). Relationship between beta-cell mass and diabetes onset.. Diabetes Obes Metab.

[21] Rahier J, Goebbels RM, Henquin JC (1983). Cellular composition of the human diabetic pancreas.. Diabetologia.

[22] Clark A, Wells CA, Buley ID, Cruickshank JK, Vanhegan RI (1988). Islet amyloid, increased alpha-cells, reduced beta-cells and exocrine fibrosis: quantitative changes in the pancreas in type 2 diabetes.. Diabetes Research.

[23] Butler AE, Janson J, Bonner-Weir S, Ritzel R, Rizza RA (2003). Beta-cell deficit and increased beta-cell apoptosis in humans with type 2 diabetes.. Diabetes.

[24] Yoon KH, Ko SH, Cho JH, Lee JM, Ahn YB (2003). Selective beta-cell loss and alpha-cell expansion in patients with type 2 diabetes mellitus in Korea.. J Clin Endocrinol Metab.

[25] Del Prato S, Marchetti P (2004). Beta- and alpha-cell dysfunction in type 2 diabetes.. Horm Metab Res.

[26] Deng S, Vatamaniuk M, Huang X, Doliba N, Lian MM (2004). Structural and functional abnormalities in the islets isolated from type 2 diabetic subjects.. Diabetes.

[27] Smolen P, Rinzel J, Sherman A (1993). Why pancreatic islets burst but single beta cells do not. The heterogeneity hypothesis.. Biophys J.

[28] Jo J, Kang H, Choi MY, Koh DS (2005). How noise and coupling induce bursting action potentials in pancreatic beta-cells.. Biophys J.

[29] Hara M, Wang X, Kawamura T, Bindokas VP, Dizon RF (2003). Transgenic mice with green fluorescent protein-labeled pancreatic β-cells.. Am J Physiol Endocrinol Metab.

